# Process Engineering Accelerating an Economic Industrialization Towards a Bio-Based World

**DOI:** 10.3390/molecules24101853

**Published:** 2019-05-14

**Authors:** Lukas Uhlenbrock, Reinhard Ditz, Jochen Strube

**Affiliations:** Institute for Separation and Process Technology, Clausthal University of Technology, 38678 Clausthal-Zellerfeld, Germany; uhlenbrock@itv.tu-clausthal.de (L.U.); ditz@itv.tu-clausthal.de (R.D.)

**Keywords:** bioeconomy, secondary metabolites, purification strategies, bio-based world, waste valorization, resource-efficient production

## Abstract

The transition towards a bio-based world is a challenging undertaking. This perspective paper, from an engineering point of view, aims to provide an overview of existing projects and academic disciplines highlighting the potential benefit of increased interdisciplinary exchanges. Furthermore, the current utilization of biomass to produce biogas is discussed, including an economic assessment, showing the need for new strategies of biomass valorization. One solution could be the development of separation processes for the isolation of secondary plant metabolites, which have been especially valuable for pharmaceutical applications, e.g., taxotere ^®^ and artemisinin. The economic feasibility is demonstrated in a case study, evaluating the purification potential of curcuminoids from *Curcuma longa* L. Subsequently, the conclusion discusses the limitations of large-scale industrial applications and the need for new separation techniques as a step towards a bio-based world.

## 1. Introduction

The reduction of the industries need for fossil resources is discussed in Germany and many other regions worldwide. Geopolitical considerations may provide additional motivation for research to substitute renewable resources for fossil fuels, as they provide independence from fossil fuels, especially for economies which are focused on agriculture. Bio-based industries on the EU level and bioeconomy in Germany represent large funding initiatives [1,2,3]. Technologies considered in principal are either “white biotechnology” or “renewable resources”, and plant-based systems, with “flow charts” existing in abundance [4], pilot scale operations being considerably less [5,6], while only a few have been realized as fully operated plants [4,7].

The range of applications is fairly comprehensive for mobility and ranges from replacement of diesel and gasoline all the way to the generation of energy via biogas, and as raw materials for the chemical and pharmaceutical industry (ChPI) instead of exhausting fossil sources [8,9,10].

Renewable resources and biotechnology are not by definition more ecological; instead a careful process technological optimization is required in combination with closing the loops (recycles) and establishing economic scenarios in globalized markets including the setup of integrated eco-balances. Otherwise, an unnecessary and fruitless emotional discussion without a sustainable outcome will result. Plants are not good per se, but also require resources for cultivation, harvesting, and processing, for example, corn can sometimes be dangerous and poisonous such as digitalis (*Digitalis purpurea* L.) and heracleum (*Heracleum mantegazzianum* Sommier et Levier). Introduction of invasive species into new environments can have a drastic environmental impact. Furthermore, excessive and unsustainable industrial cultivation of renewable resources can damage any ecosystem, e.g., clearing of rainforest for palm oil production or soil erosion from energy corn cultivation [11,12,13]. On the other hand, crude oil is not à priory bad because of its high energy density and efficient processability [14,15].

As always, much depends on how resources are handled, which products result, and what the resulting overall benefit is. For a long time, packaging materials from renewable resources in contrast to synthetic polymers were not biodegradable [16]. Biotechnology today can even compete economically for production of bulk chemicals like ethanol or acetic acid, if the internal cycles are fully optimized and closed [17]. So far, attempts to produce polymers from succinic acid or lactic acid have not been successful, and producing tires with rubber from *Taraxacum officinale* F. H. Wigg has a niche status so far and may not be further pursued [5].

Using renewable resources in cultivation for energy production, for example, energy corn for biogas plants, generates monocultures due to the area required. The ethical public discussion “container vs plate” or “energy vs plate” enforces in a first stage a complete use (or utilization) of the existing food waste, which is currently not the case, and due to the logistics by no means trivial. One approach may be the setup of “trading houses” for available sources of renewable resources [18,19]. Additionally, most specialty crops are cultivated and refined in small regions, resulting in unique waste in high quantities, for example, artichoke, wine, hops, and olives [20,21,22,23,24,25].

An approach which is more target-oriented for setting up technological support programs is similar to those employed for the development of environmental technology programs during the 1980s. This has benefited the German industry until today, even after solving the environmental issues, for example, acid rain or air quality occurring from 1960 to 1970. A key task and challenge of process technology which remains to be developed is consistent business models including an assessment of economic feasibility linked to sustainable eco-balances.

The introduction of bioeconomy can provide a chance to correct or adjust non-sustainable technological developments in the current economic system, especially in the industrialized agricultural sector. The development of methods for the valorization of waste products is exceptionally useful, for example, animal husbandry or special crops. Use of plants for energy, be it central, decentralized, or mobile, is equally sustainable (or not) as are fossil energies, e.g., coal, crude oil, or gas. Utilization of residuals and waste, or side stream valorization is so far not too widespread, however, it enjoys strong growth worldwide in Europe as well as in Germany [26,27,28,29,30,31,32].

The utilization of primary and secondary metabolites as value products is also integrated in this concept, following the basic idea of not breaking down the synthetic performance of the plants first by disintegration into small building blocks and then recombining them by fermentation, but to first extract the value compounds, e.g., amino acids, peptides, and proteins as primary metabolites, and also extract secondary metabolites, e.g., polyphenols or flavonoids [33,34]. This must not affect further processing steps, and therefore the process engineer typically falls back to natural solvents, ideally water [17,35,36,37,38,39,40].

## 2. Actors and Involved Disciplines

The current approach to bioeconomy is focused on the substitution of conventional products with sustainable alternatives. Often, process and product development are limited to one single component. This greatly reduces the potential for the utilization of side components. The access of these components requires major changes in the traditional production process and thorough knowledge of the composition of the raw material.

**Figure 1:**
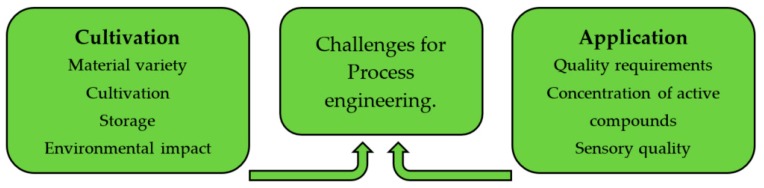
Challenges for process engineering between agriculture and application.

The function of process technology is, due to its central position within the value chain, impacted both from the application side at the end and from the materials supply side, as shown in Figure 1.

The characterization of material variability and cultivation conditions is a well-established area of interest for conventional industrialized farming, but the focus is still high yield and singular use for food production. An extended focus could also include the potential of valuable compounds, which are currently not utilized. The combination of deep knowledge about the raw material and the reinvention of traditional production processes is a main challenge for bio-economists.

Advanced approaches for the characterization of raw materials are the following current fields of research:

- Metabolic pathways of secondary plant metabolites (OMICS Technologies) [41,42];
- Growth of highly productive plants [43,44,45];
- Optimization of growth conditions for environmental impact content and yield [46,47,48,49].

The role of process technology is to subsequently maintain the quality range of the raw material and to adjust and compensate variations from environmental sources. The techniques to utilize for this purpose strongly depend on the desired product properties. Developing quality profiles of biogenic products is a substantial research area in the following contexts:

- Clarification of the metabolic profiles of secondary plant ingredients [50,51,52];
- Use of secondary plant ingredients for therapeutic application for animals and humans [53,54,55,56,57,58,59];
- Use for defense against undesired plants, bacteria, and fungi [60,61];
- Application for improving material properties.

## 3. Technological Processing of Biogenic Raw Materials

An improved value generation with simultaneously enhanced resource and energy efficiency can be reached by technologically driven processing of the plant material. As examples, the production of sugar from beet, the isolation of starch from potatoes or the extraction of hops are listed in [62,63]. These highly integrated processes allow efficient processing of up to 15,000 t raw material per day, and efficient use of energy by proper integration of waste streams [64]. In addition, the high throughput of raw material allows for efficient use of byproducts which build up during the production process. Current concepts often focus on the generation of energy from biomass, neglecting the potentials of secondary plant ingredients [4]. 

An extension of this production technology to other processes, e.g., processing of *Curcuma longa* will allow higher process efficiency as well as access to new products, which cannot be recovered by traditional means.

**Figure 2:**
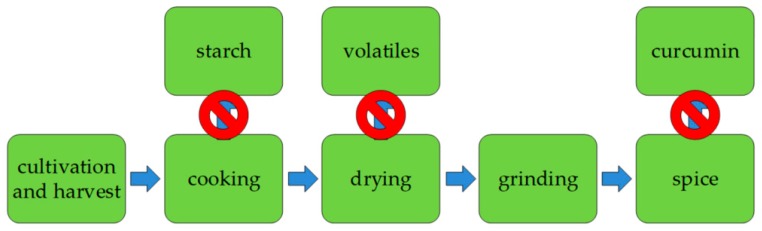
Loss of potential products by the restricted focus on traditional process technology.

The traditional processing path that focuses on the production of turmeric is marked in Figure 2 by the blue arrows [43,65]. Cooking of the rhizomes prevents recovery of starch, which is present in *Curcuma longa*. The subsequent drying step, which often takes place in the open in small scale production facilities, is why etheric compounds with interesting properties get lost [66,67]. The final product, Curcuma powder, contains, among others, the compound group of curcuminoids which are a candidate for various medicinal therapies [68,69]. This process could be a prime example, where reinvention of the traditional process could make additional products accessible. This phenomenon can be observed in many industries. 

Additional to cultivation costs and costs for logistics and marketing, the production costs are important for the economic success of a product. These costs are highly dependent on the source of the resource and the required level of final purification. The comparison of sugar (0.4 €/kg) with the price of commercially available hawthorn extract (450 €/kg) shows the cost efficiency of sugar processing compared to the processing of medicinal plants [70,71].

Different separation techniques that are in use for enhancement processes and have been developed for applications in the pharmaceutical field are:

1. Extraction is the classical separation method for secondary plant ingredients from the plant matrix. Many extracts are used in the medicinal area and defined by monographs [24,27,72,73,74,75,76,77,78,79].
2. Distillation allows the recovery and reuse of the extraction solvent and a separation based on the boiling points of the individual components. The solvent free residues are sold as dried extracts or further purified [80].
3. Liquid-liquid extraction of a liquid extract with a second, non-miscible solvent can be used to further reduce the side-component spectrum of the product. Depending on the operation of the extraction, the product purity can be further enhanced [40,81].
4. Adsorption and chromatographic separation techniques allow the purification of complex mixtures. These techniques, in particular, are applied to the separation of pure compounds [72,82].
5. Crystallization is a separation technique which allows a separation of compounds based on their melting point or solubility [39,83].

In addition, mechanical separation processes (sieving, air separation and electrostatic separators) can be used for selection and classification of raw materials.

These separation techniques have been developed and adopted to produce high value products for a long time. Significant improvements regarding process robustness and costs make them the tool of choice to isolate new products from traditional processes, like the process shown in Figure 2.

## 4. Value-Added Products from Sustainable Raw Material

The potential of secondary plant ingredients as a basis for value generating products is high. Especially well-known are plant ingredients occurring in food and contributing to the positive impact of healthy nutrition [84,85]. Especially in agricultural regions and countries the necessary work up of the material is performed by the farmer [86,87]. The processing of single harvests often does not allow the utilization or work up of waste streams, as they occur in too small amounts to permit economical extraction. 

The potential of secondary plant ingredients is, however, not limited to health promoting effects from eating the plant of a respective compound. Often the positive potential of the respective compound cannot be utilized by the intake of the ingredients alone, because the concentration is often not more than 1% of the dried raw material, or bioavailability is not sufficient [44,87,88,89]. In addition, many plants often generate not only one form of a compound, but different forms with varying properties. The low concentration of these compounds in the raw material prevent an economic isolation of the materials in the processing range of 50 tons raw material per year, which is the typical range for small and medium enterprises in the extraction. 

The comprehensive use of process technology for the use of biogenic raw materials is well established up to a scale of sugar production. These processes allow processing of plant material in the range of several 10,000 tons per day [90,91]. Contrary to primary plant ingredients, as in the case of sugar and starch, secondary plant ingredients are often present in much smaller concentrations in the raw materials, as shown in the following tables. Many concepts for the development of biorefineries focus on primary plant ingredients for production of materials or energy carriers with a selected number of examples are listed in Table 1 [92,93,94,95].

The sources of raw material compete with traditional food supply, which also contains active ingredients [98]. Only further processing allows collection of products in enough concentration for identification of a therapeutic effect. In the case of *Curcuma longa*, processing turns the starch present into a paste-like consistency during cooking and as such cannot be isolated and utilized, even though starch represents approximately 20% of the fresh mass of *Curcuma longa* roots [99].

Currently, traditional herbal medicinal products do not generally need to be provided as pure compounds. Efficacy of many plant-based products is recognized by EMA (European Medicines Agency) and manufacturing of plant-based pharmaceuticals is laid down in monographs [100,101,102]. However, the regulatory agencies have shown growing interest in the identification of side components and their effect on the product efficacy. High value products listed in Table 2, e.g., 10-DAB III (taxotere^®^), which is used for the treatment of various cancer types or artemisinin and for the therapy of malaria, require high purity. The cost of generating or improving a biogenic product strongly depends on the level of purification [78].

### 4.1. Requirements for the Design of Processing Plants to Recover Secondary Plant Ingredients

Process development does not stop with the identification of useful components or the design of resource efficient separation processes.

**Figure 3:**
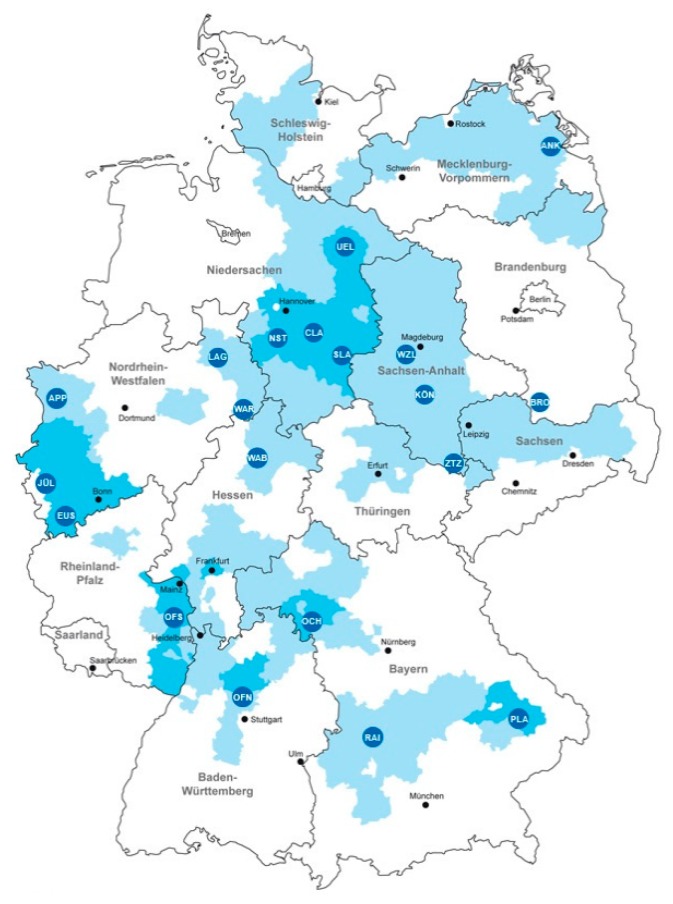
Cultivation area of sugar beet and location of processing plants [107].

The logistics of the raw material is an important element of the value chain. The use of biogenic resources has specific requirements for the technical implementation in manufacturing plants. Already, many value products containing plants degrade shortly after harvest and have to be immediately either dried or processed. The example of sugar processing shows that the raw material is spread over large areas, as seen in Figure 3. [105]. Modern plants for sugar refining are designed to handle more than 10,000 tons per day. 

**Figure 4:**
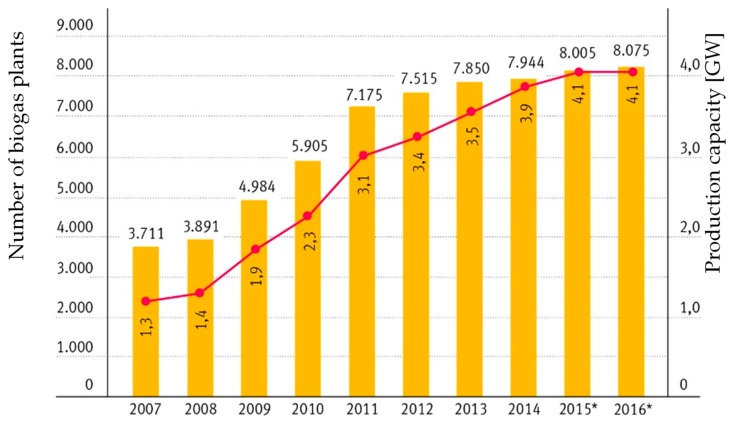
Number of plants (left axis) and corresponding production capacity in Gigawatts (GW) (right axis) of biogas plants in Germany, from 2007 to 2016 [108].

However, for the secondary plant ingredients in pharmaceutical or cosmetic applications amounts of less than 100 tons of raw material per year need to be processed, and therefore require only small plants, which allow for provision of a high level of added value. The feasibility of implementing such a decentralized infrastructure is demonstrated by the development and deployment of Biogas plants in Germany from 2007 to 2015, which is shown in Figure 4. During this time, approximately 8000 plants were installed in order to produce biogas from biomass [106].

### 4.2. Economic Assessment of Biogas Generation

**Figure 5:**
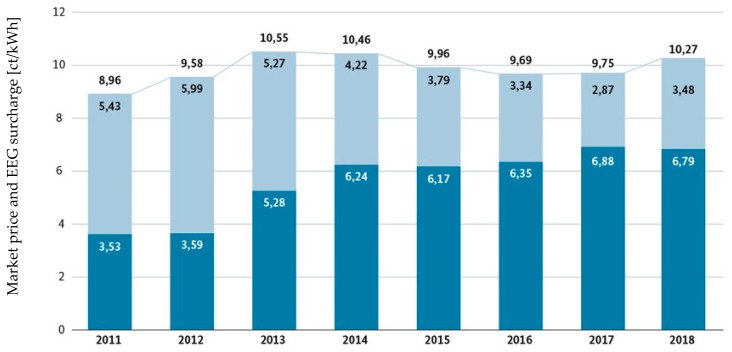
Sum of market price for electricity and EEG surcharge 2011–2018 [112].

The generation of biogas is an interesting field from an engineering point of view. The utilization of decentralized, small-scale production plants could be one solution to the logistical challenges described above. Unfortunately, biogas generation is under increasing economic pressure due to declining funding/subsidies and declining energy prices. This pressure becomes especially evident when comparing price development for electricity over the last years. As can be seen in Figure 5, the price between 2011 and 2018 fell 35% to 3.48 ct/kWh. The comparable call for electricity from biomass plants required to be run renewable energies since 2017 has a median price of 14.20 ct/kWh [109,110].

The same call for electricity from wind farms in February 2018 has resulted in an average current price of 4.73 ct/kWh. This is significantly below the market price for power from biogas. With the high compensations on old supply contracts from biogas plants running out, it is questionable, whether all existing biogas plants can still operate profitably in the future [108]. Biogas plants, which rely to a high degree on energy crops as feedstock, see the highest economic pressure. 

The use of biomass with the primary goal of electricity production must be reconsidered. The German biomass research center (DBFZ) expects a decline in biogas plants to continue until 2030 [111]. 

A network of decentralized processing plants for production of secondary plant ingredients, comparable to biogas plants provides access to additional products, enabling a better utilization of the plant-based raw material. Subsequently, the extracted plant residues can still be transferred into energy.

### 4.3. Production of Necessary Raw Materials

**Figure 6:**
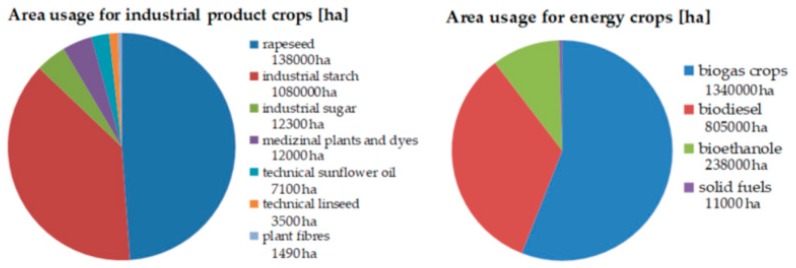
Area usage for industrial and energy crops in Germany in hectares [114].

Examples of renewable resources used by industry are sugar, plant-based oils, and starch. They are cultivated in Germany on a total area of 270,000 acres, equivalent to a share of 2.4% of the total available agricultural area (Figure 6, left side) [113]. This is in comparison to an area of 2.39 million hectares for the generation of biomass, which is primarily used for energy purposes (Figure 6, right side). Approximately 1.34 million ha, 11.37% of the German agricultural area, are allocated to grow energy plants for biogas production according to data from FNR and BMEL [114].

The economic pressure on biogas production can reduce demand for these crops, which has the opportunity to establish new cultivations with additional uses. In the value chain for the energetic use of biomass, no utilization of the secondary metabolites of feedstock is considered. However, a successful implementation of a bio-economic model depends on an optimal utilization of efficient plant ingredients, if they want to provide an alternative to established products. 

Therefore, the growth and direct use of energy plants not only directly competes with food production, but, in addition, leaves a large potential for secondary plant ingredients to be unused. In addition, growing biomass for the isolation of secondary plant ingredients, such as curcumin or others, allows for further energetic use of the biomass, which is a common practice in sugar production [107]. It must be kept in mind, that the biomass yield of plants, producing valuable secondary metabolites, is typically lower than that of optimized energy plants. The impact on the electricity generating community in Germany, however, is minimal due to the small share of biomass based in energy generation [115]. Nevertheless, the energetic use of biomass through biogas generation remains to be a useful technical application of renewable energy [116].

The generation of biomass is labor- and cost-intensive, resulting in high prices. The contribution margin, for example potatoes in Germany, is in the range of 2000–2500 €/ha, with potato production being a well-established process using specialized equipment and optimized procedures [117,118]. Other field crops such as sugar beet, corn, and alternative substrates for biogas generation are calculated with contribution margins in the range of 1000–1200 €/ha [119,120,121]. Depending on the condition of the plant material during processing, the processing costs, as well as the logistics between the field and the processing plant, need to be added to the contribution margin. These additional costs can be significantly higher than the contribution margins. As an example, the full cost for the cultivation of potatoes is calculated as 4600 €/ha, which is approximately twice the contribution margin [118].

As cost calculation is correlated to the area in use, for the estimation of raw material costs of a biomass processing plant with the goal of producing secondary plant ingredients, the revenue per area for the raw material processed must be assessed. A selection of yields for field crop is shown in the following Table 3.

### 4.4. Economic Potential of Process Technologically Enhanced Products on the Example Processing of Curcuma longa

*Curcuma longa* belongs to the family of Zingiberaceae, and in particular, in Asian countries, it is grown as a spice plant [74]. The rhizomes of *Curcuma longa* contain 3–5% curcuminoids as well as 2–7% etheric oils and starch [123].

Curcuminoids possess a variety of properties, which have been investigated and are of interest for medicinal applications [75,124,125,126]. Traditionally, the rhizomes of the *Curcuma longa* are cooked, dried, and ground after harvest by the farmers [127].

If the cultivation of *Curcuma longa* is comparable to the cultivation of potatoes, similar full costs in the range of 5000 €/ha, as cited above, can be assumed. Together with an estimated yield of 50 t/ha, as reported in the literature cited in Table 3, the cost of the raw material is approximately 100 €/t. 

The following three scenarios serve as a starting point for the evaluation of the process concepts:

1. Traditional two people farm, plant capacity 0.2 t/d, low overall efficiency (wood-based energy generation, no energy recovery);
2. 100 worker production facility with 25 t/d capacity, average efficiency (oil-based energy supply, simple energy recovery);
3. Large-scale processing such as sugar plant (integrated power plant, efficient energy recovery and utilization).

The process concepts are based on published studies in laboratory scale as well as reports on generation and processing of *Curcuma longa*. Investment costs are assessed in a class 5 cost approximation, based on the processing scheme and the costs of known plants [128,129].

The concepts are summarized in Table 4, showing the production capacity and catchment area of each scenario, together with the different assumptions regarding fuel source and efficiency. 

The classic processing of *Curcuma longa* markets curcuma spice as a product. The traditional processing is done by the farmers who grow and harvest the plants, as well as process the harvest and sell the final product.

This decentralized production described in scenario one uses all available resources, i.e., wood for heating the kettles. Therefore, resource efficiencies for these processes are quite low, while the corresponding manual labor is quite high. Scenario two can be visualized as a farmer collective, utilizing mechanized cultivation and harvesting to improve energy efficiency and productivity. Current approaches to access the curcuminoids use the curcuma spice as the raw material for isolating the pure compound. Therefore, the production of curcuma spice is included in the economic considerations.

**Figure 7:**
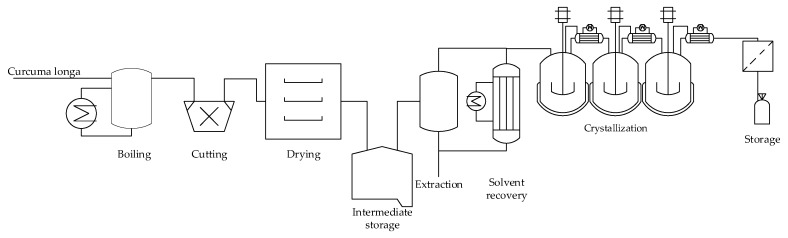
Small and medium scale extraction process for purification of curcumin.

The process consists of the steps which are shown in Figure 7. In the first step the fresh raw material is cooked, whereby, the starch in the root agglomerates and this improves the milling properties [43]. Then the material is dried. The dry material is extracted with ethanol. From the extract the curcumin can be crystallized. 

The alternative, large-scale approach considered in scenario three allows collection of the starch fraction and the etheric components of *Curcuma longa*, in addition to the main product curcumin, during processing. These product fractions are lost during the cooking and drying of the traditional processes, as described in scenario one and two. 

**Figure 8:**
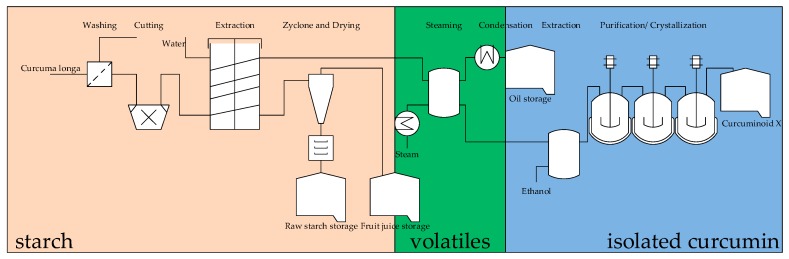
Large-scale process for the extraction of starch, etheric components, and curcumin.

Figure 8 represents the schematics of the large-scale production process which was derived from the sugar processing process.

**Figure 9:**
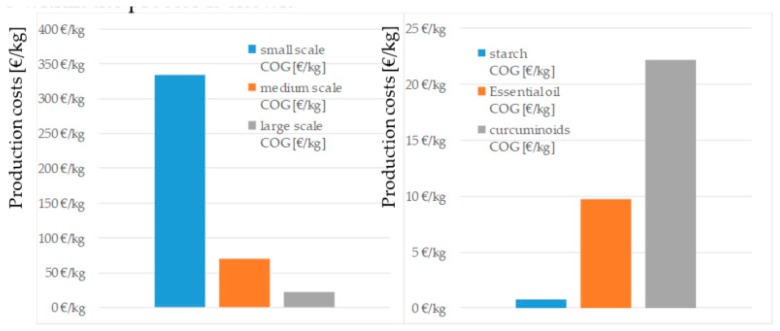
Comparison of production costs for the different scales (left) and distribution of production cost by product of scenario three (right).

Looking at the cost of the process in Figure 9 it can be concluded that overall costs decrease with increasing scale of the process, which is the typical effect of economy of scale. This cost decrease stems from an improved energy efficiency, higher plant utilization, and lower specific investment costs, and it is reflected in a decrease of the specific production cost by a factor of five, which is shown on the left side of Figure 9. Better utilization of the raw material allows for the redistribution of production cost to additional products, such as starch and the volatile fractions, whereby, the production cost of curcumin can be reduced by a factor of three. On the right side of Figure 9, the distribution of the production cost in scenario three within the process is shown.

**Figure 10:**
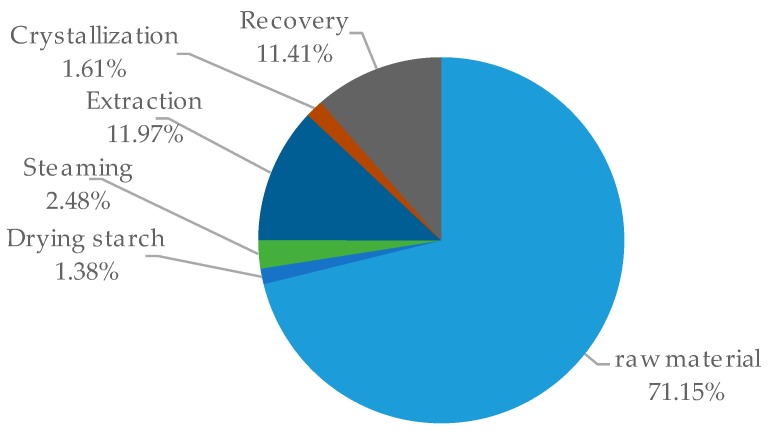
Distribution of operating costs.

The annual costs of operation are shown in Figure 10. The major share of the operating costs, which is around 70%, is associated with the acquisition of the raw material. This distribution is typical for the processing of renewable resources, highlighting the importance of cost-efficient production processes. Other major contributions to the total operating costs are extraction and solvent-recovery. The extraction costs are driven by replacement, which is assumed to be 10% of solvent volume per year. Solvent recovery is cost intensive because it requires large amounts of energy for vaporization and condensation.

Assuming a share of 70% for distribution, logistics, and taxes, as well as a desired return on investment (ROI) of 3 years, the following prices for the three considered scenarios are found to be:

- Scenario 1 price of curcumin is 1475 €/kg;
- Scenario 2 price of curcumin is 310 €/kg;
- Scenario 3 price of curcumin is 187 €/kg, essential oil is 30 €/kg starch is 1 €/kg.

**Figure 11:**
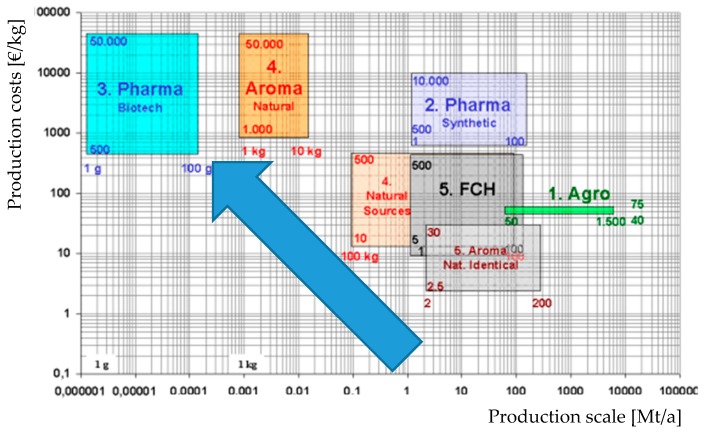
Dependency of production costs on the target market [130].

The most obvious conclusion from these considerations can be reached by comparing these prices with of the products displayed in Figure 11. This comparison shows that cost-efficient production of secondary plant metabolites is possible in every scale, for medium to high-end products, i.e., aroma or pharma applications. 

Especially, processes that have a high throughput for the low-price segment, for example sugar and starch, could profit from additional products which would compensate for the high raw material costs and increased resource utilization. The isolation of secondary metabolites with an average content of 0.1% and with 50% yield from the raw material of a typical sugar production plant, cf. Table 4, would amount to 890 tons of product per year. On the other hand, medium scale production, which focuses on the isolation of high value products, as seen in scenario two, could produce an additional amount of 160 tons of primary metabolites, with an average content of 10% and 50% yield.

It must be kept in mind, in order to use specific curcuminoids for a pharmaceutical preparation further costs arise for final purification and formulation. The additional efforts and the subsequent significantly increased production costs are offset by the higher value of the final product.

## 5. Summary and Conclusions

Decentralized and small-scale production solutions for agricultural communities is a way to produce new and renewable products. The purification of isolated structures from plants will be of increasing importance in the future. Innovative technologies create optimal COGs [35], especially, process intensification such as hybrid separation technologies or integration of reaction and separation, for example phyto-SMB (simulated moving bed) chromatography processes may be a solution. 

**Figure 12:**
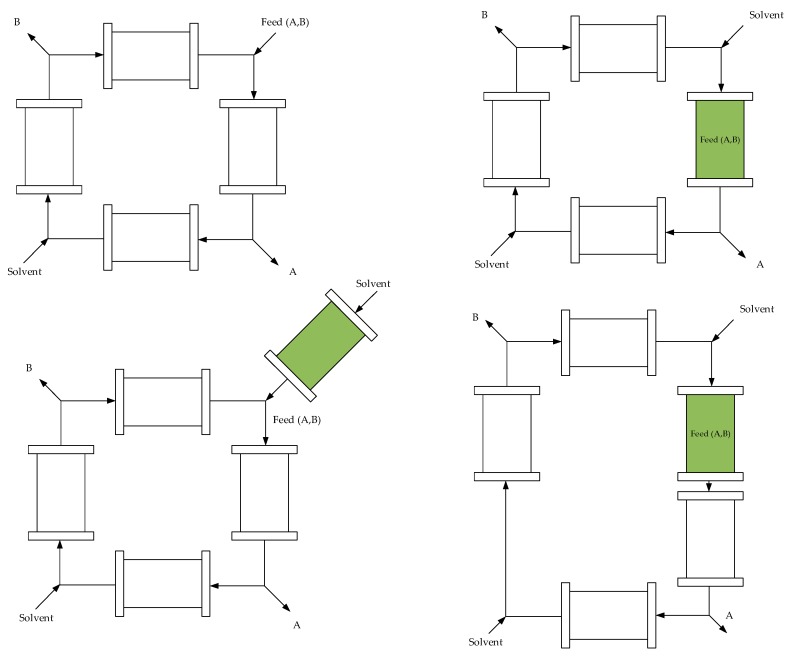
Potential configurations of Phyto-SMBC, upper left: conventional SMBC, all other flow charts depict potential process options of Phyto-SMBC processes, plant raw material in green with component faction A strong binding in chromatography as extract and component faction B as weaker binding raffinate.

Counter-current processing, as depicted in Figure 12 efficiently utilizes most resources such as adsorbent and solvent amount, and in addition, integrating separation and reaction is a well-known tool for process intensification [131,132,133,134,135,136,137].

The value chains of plant-based products can be extended by the extraction of secondary plant metabolites which requires efficient separation techniques.

Current concepts for use, such as the energetic use of biomass, must be reconsidered, because the potential of primary and secondary contents are not utilized/exploited. The high cost of farming and the limited available farming area makes a more intensive use of the available raw materials that are indispensable for the progress of a bio-based economy. Nevertheless, the generation of energy from biomass is still a viable option for the valorization of waste streams from industrialized farming, e.g., manure from animal husbandry. The current usage of energy crops should be reconsidered. In light of the overproduction of crops for food production, alternative crops and economic products are needed to provide an incentive for the cultivation of currently uncommon crops. An increase in scientific work is needed to provide new materials and technological solutions for the manufacturing of such products. 

First, examples for new products and alternative feedstocks are already available, but require additional optimization to become competitive, while maintaining sustainability. One approach is the substitution of conventional solvents with solvents made from biomass, e.g., Pinen, 2-Methl THF, vegetable oils and others. Additionally, new packaging materials could provide a more sustainable alternative to conventional polymers and provide use for currently unused waste stream. This approach has been expanded to bulk chemicals, e.g., alternatives to rubber from dandelions. Examples of new products from renewable resources are food additives, such as amino acids, peptides and proteins. The development of new techniques for the refinement of natural raw materials could also provide new value products, for example flavonoids, polyphenols and aroma. 

The example of the generation of curcumin from *Curcuma longa* demonstrates the limitation of resources used by traditional production technology with a focus on single products and the potential of the expansion of existing production processes. At the same time, the development of large-scale processes, as shown in scenario three for the example of a plant size, the scale of a sugar plant, is not reasonable without limits, as very large areas are required for supplying the raw materials. The efficiency which is increases by the improved process technological handling suffers from the strong increase of the necessary logistics, and therefore it loses its economic impact. 

The concept of large-scale biorefineries cannot simply rely on economy of scale, because of the area requirements of agricultural crops. A possible alternative as a compromise between processing efficiency and logistics is the development of efficient processing plants with capacities in the range of 5000 tons per year, which could be supplied by local agricultural associations. 

## Figures and Tables

**Table 1:** Industrially produced primary plant ingredients.

Plant Material	Content	Quantity	Source
Sugar beet	Saccharose	18% of fresh mass	[70]
Potato	Amylose, Amylopektin	15–20% of fresh mass	[96]
Wood material	Lignin	20–30% of fresh mass	[97]

**Table 2:** Presence of secondary plant metabolites.

Plant Material	Active	Concentration	Product	Source
*Artemisia annua* L.	Artemisinin	0.4% TM	pharmaceutical	[103]
*Piper nigrum* L.	Piperin	6.5% TM	nutrition	[104]
*Taxus baccata* L.	10- DAB III	0.3–0.7% TM	pharmaceutical	[78]
*Curcuma longa* L.	Curcumin	3–5% TM	nutrition	[43]

**Table 3:** Agricultural yield for selected field crop.

Plant Material	Fresh Mass Yield	Utilized Yield	Source
Sugar beet	76 t/ha	n.a.	[122]
potato w/o greens	45 t/ha	n.a.	[122]
St. John’s wort	20 t/ha	7.5 t/ha	[45]
Wheat plant with grain	26 t/ha n.a	n.a. 7.6 t/ha.	[122] [122]
Stinging nettle	26.4 t/ha	2.7 t/ha	[45]
Curcuma without greens	17–60 t/ha	n.a.	[43]

**Table 4:** Key parameters of the assessed production scenarios.

Key Parameters	Scenario 1	Scenario 2	Scenario 3
Processing capacity/a	25 t/a	3200 t/a	1,782,000 t/a
Catchment area	0.5 ha	64 ha	35,640 ha
Energy efficiency	10%	30%	80%
Fuel	wood	diesel	coal
Product/manufacturer	0.01 t/a	22 t/a	13,000 t/a
Employees	2	80	130
Products	curcuminoids	curcuminoids	curcuminoids starch essential oil
